# Notch Signaling in Cardiovascular Disease and Calcification

**DOI:** 10.2174/157340308785160552

**Published:** 2008-08

**Authors:** Gabriel Rusanescu, Ralph Weissleder, Elena Aikawa

**Affiliations:** Center for Molecular Imaging Research, Massachusetts General Hospital, Harvard Medical School, Boston, MA, USA

**Keywords:** Calcification, cardiac valve, inflammation, Notch signaling, mesenchymal stem cells, atherosclerosis.

## Abstract

Recent increase in human lifespan has shifted the spectrum of aging-related disorders to an unprecedented upsurge in cardiovascular diseases, especially calcific aortic valve stenosis, which has an 80% risk of progression to heart failure and death. A current therapeutic option for calcified valves is surgical replacement, which provides only temporary relief. Recent progress in cardiovascular research has suggested that arterial and valve calcification are the result of an active process of osteogenic differentiation, induced by a pro-atherogenic inflammatory response. At molecular level, the calcification process is regulated by a network of signaling pathways, including Notch, Wnt and TGFbeta/BMP pathways, which control the master regulator of osteogenesis Cbfa1/Runx2. Genetic and *in vitro* studies have implicated Notch signaling in the regulation of macrophage activation and cardiovascular calcification. Individuals with inactivating Notch1 mutations have a high rate of cardiovascular disorders, including valve stenosis and calcification. This article reviews recent progress in the mechanism of cardiovascular calcification and discusses potential molecular mechanisms involved, focusing on Notch receptors. We propose a calcification model where extreme increases in vascular wall cell density due to inflammation-induced cell proliferation can trigger an osteogenic differentiation program mediated by Notch receptors.

## CARDIOVASCULAR CALCIFICATION IS AN INFLAMMATORY DISEASE

Cardiovascular disorders are the most prevalent cause of death in the US. Atherosclerosis, the most frequent cardiovascular disorder, is the result of a gradual accumulation of atheromatous plaques in the arterial wall. The prognostic is especially unfavorable when such plaques accumulate in the coronary arteries, cerebral arteries or aorta, where they can lead to often-fatal cardiovascular accidents such as stroke, heart attacks or heart failure. Plaque calcification causes an increased risk of rupture due to mechanical stress at the interface between the rigid calcified plaque and neighboring soft “unstable” atheroma [1-3]. In addition, microcalcification in the thin fibrous cap may cause microfractures leading to plaque rupture, thrombosis and acute cardiovascular events [4]. Furthermore, calcification impacts clinical outcome not only by complicating atherosclerosis but also by impairing the movement of aortic valve leaflets, increasing arterial stiffness or causing plaque fracture during angioplasty.

A number of defects and/or polymorphisms of proteins involved in the fat transport pathways, notably the apolipoproteins and the LDL receptor, markedly contribute to increased atheroma and calcification risks. Defects in these proteins are associated with hyperlipoproteinemia or hypercholesterolemia, have a frequency of 1:500 to 1:1000 in the general population and are characterized by a significant increase in coronary heart disease susceptibility. In the past decade however, preclinical and clinical studies have proposed that active osteogenic mechanisms contribute to arterial and valvular calcification, replacing the traditional view of passive lipid accumulation and cellular degeneration. This idea has only recently started to be accepted and investigated, although the underlying information has been available for several decades. Early *in vitro* studies proposed that calcification is the result of an inflammatory process induced by LDL oxidation and uptake by macrophages, which become foam cells [5]. This model was supported by *in vivo* results showing that anti- inflammatory drugs such as statins may also inhibit osteogenic pathways in myofibroblasts of hypercholesterolemic rabbits [6-8]. Recent advances in signal transduction have expanded this model, networking together a number of signaling pathways previously regarded as unrelated. A simplified diagram of the processes involved in calcification, associated with corresponding molecular signaling mechanisms, is shown in Fig. (**1**).

Our recent studies have used innovative functional imaging strategies to monitor valvular and atherosclerotic lesion initiation and progression. These have provided robust evidence that inflammation correlates with osteoblastic activity preceding the development of advanced calcification*, *suggesting that inflammation triggers osteoblastic activity of myofibroblast-like cells thus promoting calcification (Fig. (**2**)) [9-12]. Furthermore, we also demonstrated that statin treatment of early-stage atherosclerosis reduced inflammation and osteogenic activity in parallel, supporting the concept that early pharmacological modification of proinflammatory processes retards the progression of cardiovascular calcification [12]. 

While intimal calcification is connected to the atherosclerotic process and lipid processing, type II diabetes and chronic kidney disease often lead to the calcification of the medial layer of the vascular wall. This process is connected to elastin degradation by proteolytic enzymes rather than to lipid transport and oxidation and can take place independently of atherosclerosis. Elastin degradation peptides have pro-inflammatory properties similar to the oxidized cholesterol and can act as macrophage chemoattractants [13] (Fig. (**1**)). Genetic defects that result in elastin fiber degradation (Williams-Beuren syndrome, pseudoxanthoma elasticum, Marfan syndrome) have been associated with fibroblast and smooth muscle cell proliferation, cardiovascular calcification as well as with a chronic inflammatory state. Although having different etiologies, both intimal and medial calcification involve the activation of a pro-inflammatory mechanism and the initiation of smooth muscle cell proliferation, which likely continue through similar calcification paradigms. A very similar thickening and calcification process induced by inflammation affects heart valves [14, 15], leading to valve stenosis, characterized by decreased valve elasticity and incomplete closing/opening, and to calcific aortic valve disease that currently has no therapeutic options other than surgical valve replacement.

This review discusses recent developments in unraveling the cellular and molecular aspects of cardiovascular calcification as a key process in cardiovascular disease, focusing on the Notch pro-inflammatory pathway as a candidate target for pharmacological therapy with the potential to replace surgical solutions.

## CELLULAR MECHANISMS OF CARDIOVASCULAR CALCIFICATION

The elucidation of the mechanisms of cardiovascular calcification is an ongoing process, complicated by the fact that even the origin of calcified cells in cardiovascular lesions is yet an unresolved question. Osteoblasts may arise either through vascular smooth muscle cells (VSMC) de-differentiation and proliferation [16], from circulating bone-marrow-derived stem cells in the blood [17, 18] or from multipotent calcifying vascular cells resident in the vascular wall that have the potential to differentiate into VSMC, osteoblasts and chondrocytes [19]. All these mechanisms are likely to contribute to some degree, although the repertoire of cell lineages present in lesions and their capacity for self-renewal suggests that mesenchymal stem cells, either resident or bone-marrow-derived, have a significant contribution. An *in situ* source of mesenchymal stem cells can be also provided through endothelial-mesenchymal transition, a mechanism that occurs in cardiac valves throughout embryonic development and disease [20, 21]. Establishing the trigger, origin and mechanism of osteoblast and VSMC proliferation are important steps in elucidating the sequence of cardiovascular calcification. However, the *in vivo* identification of mesenchymal stem cells as osteoblast precursors has been a notoriously difficult task due to their very flexible phenotypes. Although a large number of cellular markers have been used to identify mesenchymal stem cells, such as Stro-1 [22], CD105, SB-10, these are also expressed in some cells of mesenchymal origin and therefore cannot unequivocally identify stem cells to determine their location or frequency. The only accepted method to establish the mesenchymal stem cell phenotype is to demonstrate their pluripotency and regeneration over a large number of cell divisions. An indirect method used to infer the presence of mesenchymal stem cells was to demonstrate that allograft blood vessels contain in the neointima surrounding graft lesions smooth muscle cells derived from the host, not from the donor [17, 18].

Similarly to the origin of osteoblast and smooth muscle cell precursors in the neointima, the mechanism by which the inflammatory process triggers the proliferation of these precursors remains unclear as well. Macrophages can induce osteogenic differentiation and calcification both through direct cell contact with precursor cells or by releasing signaling molecules such as 1,25-dihydroxyvitamin D (calcitriol) (Fig. (**1**)). This hormone promotes VSMC calcium influx and calcification by interacting with the Wnt pathway [23] at the same time it promotes osteoclast and osteoblast activation and bone remodeling. Macrophages can also release pro-inflammatory molecules such as TNF-alpha (see below). Another possibility is that direct intercellular contact between activated macrophages and osteoblast precursors may also activate an osteogenic program by juxtacrine signaling, for example through Notch-mediated interactions (Fig. (**1**)). 

Blood vessels need to maintain their ability to respond to injury by repairing damaged blood vessels and sprouting new ones, therefore cells involved in this process such as VSMC and endothelial cells are not terminally differentiated and retain some ability to respond to inflammatory signals by triggering cell proliferation. In case of inflammatory signals triggered by oxidized LDL, VSMC accumulate in the neointima instead of extending new blood vessels. This dramatic increase in cell density and intercellular contact is likely to activate signaling pathways that are normally quiescent, including the proliferation of any resident mesenchymal stem cells [24]. Although the role of mesenchymal stem cells in cardiovascular calcification is still under debate, they have been frequently used as a model for calcification due to their ability to differentiate into osteoblasts and reproduce osteoblast calcification. We find that during cultured mesenchymal stem cell calcification, highest cell density areas are the first to express alkaline phosphatase (ALP), a typical early calcification marker, then form calcified nodules similar to *in vivo* cardiovascular calcification*. This suggests that excessive neo-intimal proliferation of VSMC associated with arterial stenosis may similarly initiate osteogenic trans-differentiation pathways *in vivo* through mechanisms discussed below, which may involve an abnormal activation of Notch receptors or integrins. In turn, these receptors can further over-stimulate VSMC proliferation in a positive feedback loop. In parallel, Notch ligand Jag1 may reduce control over cell proliferation by overriding cell contact inhibition, contributing to an additional increase in cell density [68].

A concurrent mechanism of calcification following the proliferation of a large number of smooth muscle cells and reduced nutrient and oxygen access to cells due to tighter cellular packing may be mediated by hypoxia and nutrient deprivation. This can explain why calcification nodules usually appear inside the neointima, in highly proliferative areas, and then extend towards the arterial lumen [21]. In fact “high-risk” plaques are primarily characterized by high levels of inflammation, not by the magnitude of arterial occlusion [25]. Hypoxia can activate signaling pathways similar to those involved in inflammation, particularly the expression of hypoxia-inducible factor (HIF-1, Fig. (**1**)), a transcription factor that promotes cell survival in hypoxic conditions typically associated with accelerated cell proliferation, for example in cancer, atheromas or proliferating stem cells (reviewed in [26]). HIF-1 plays a central role in the inflammatory response associated with hypoxia and macrophage invasion, in the angiogenesis induced to offset the effects of hypoxia [27] and in osteogenesis [28], all of which are conspicuously present in atheromas. In addition, HIF-1 binds to Notch directly and acts as a co-activator for CSL-mediated transcription [29], providing a direct link between inflammation, Notch signaling and calcification (Fig. (**3**)). 

## SIGNALING MECHANISMS IN CARDIOVASCULAR CALCIFICATION

The list of proinflammatory factors that regulate osteogenic differentiation of vascular cells is rapidly growing and includes: osteopontin, BMPs, Msx-2, oxidized lipoproteins, RANKL, PTH, matrix GLA protein, fibrillin, pyrophosphate, tumor necrosis factor-alpha, oxysterols, osteoprotegerin, insulin-like growth factor, interleukins, 1,25-di-hydroxyvitamin D, transforming growth factor-beta, estradiol, decorin, fetuin, and many others. Following the initial cardiovascular injury and inflammation, which may be caused by a number of factors such as high blood cholesterol, infections or genetic disorders, calcification likely proceeds through a unique molecular mechanism that involves the concerted action of a complex signaling network which is highly similar to normal skeletal bone development.

One signaling pathway directly connected to the lipid transport system with a well-established role in osteoblast differentiation and calcification is the Wnt pathway (Fig. (**1**)). Wnt signaling includes LDL receptor related proteins Lrp5/6, which are co-receptors for Wnt binding but can also bind ApoE [30]. Therefore ApoE downregulation has a two-fold effect on cardiovascular calcification: it results in reduced lipid clearance from the blood, thereby enhancing cholesterol deposits in blood vessels, but also eliminates the competition for Wnt receptors Lrp5 and Lrp6, effectively resulting in an activation of the Wnt pathway. The key role of Wnt as an inducer of osteoblast differentiation and calcification is mediated by beta-catenin, which induces the expression of Runx2/Cbfa1 [31], considered a master regulator of osteogenesis [32]. Runx2/Cbfa1 knockout mice die soon after birth and show complete absence of ossification. Runx2/Cbfa1 initiates a sequence of calcification regulators such as osterix (Osx) [33] , Msx (homeobox-7) [34, 35] and alkaline phosphatase (ALP), which are early markers of calcification, and osteocalcin, a marker of osteoblast differentiation [32, 36] (Fig. (**1**)). Wnt implication in calcification is part of a larger signaling network that also includes BMP [37], TGFbeta, Notch [38] and Hedgehog. These pathways interact with each other at multiple levels that are just beginning to be understood.

While Wnt signaling is thought to promote osteoblast differentiation and calcification, Notch is known to promote cell proliferation and activation. Wnt and Notch cellular signals complement each other in an oscillating network of signals associated with a segmentation clock that regulates embryonic somite development [39]. The two pathways may recapitulate a similar process in the adult cardiovascular system when triggered by injury signals that induce quiescent adult stem cells, VSMC or valvular myofibroblasts first to proliferate and then differentiate into cells necessary for tissue repair. Wnt and Notch pathways interact at several levels through positive and negative feedbacks [40]. Wnt induces the expression of NUMB, a Notch inhibitor that promotes cell differentiation, but also the expression of Jag1, an activating ligand of Notch (Fig. (**1**)). In turn, Notch signaling induces the expression of Dkk2 (Dickkopf), a Wnt signaling inhibitor thought to have osteoclastogenic and tumor suppressor properties [41, 42]. Hey1, another Notch target, is thought to inhibit osteoblast differentiation by suppressing Runx2/Cbfa1-mediated transcription [38]. The Notch pathway has been extensively studied in conjunction with its role in cell fate determination in the nervous system [43], but only recently has the picture of Notch implication in cardiovascular calcification started to emerge. The recent discovery that Notch1 inactivating mutations lead to severe heart disorders, including valve defects and calcification [44], has amplified research efforts in this direction. 

## NOTCH SIGNALING

Notch signaling is a complex juxtacrine signaling mechanism initiated by the interaction of Notch transmembrane receptors (Notch1-4) with their ligands, which are also transmembrane proteins (Fig. (**3**)). Notch ligands include “classical” ligands Jagged – Jag1 and Jag2 - and Delta-like - DLL1, DLL3 and DLL4 - as well as several atypical ligands DNER [45], F3/Contactin1, NB-3/Contactin6 [46] and Delta-like 1 homologue (Dlk1). Notch receptors and classical ligands have one DSL and numerous EGF-like domains present in their extracellular region, while atypical ligand Dlk1 only contains 6 EGF-like repeats and acts as a negative regulator by competing against classical Notch ligands [47]. The different types of ligands initiate different signaling pathways for Notch receptors summarized in Fig. (**3**). Notch receptor selectivity for either DLL or Jag ligands is regulated through glycosylation by Fringe glycosyltransferases [48]. Binding of ligands on “sending cell” surface to Notch receptors located in “receiving cell” membrane results in sequential cleavage of Notch receptors on the extracellular and intracellular sides of cell membrane (designated as S2 and S3) by the TACE/ADAM family of metalloproteases and the gamma-secretase complex, respectively. Receptor cleavage releases the Notch intracellular domain (NICD), which translocates to the nucleus and converts the CSL transcriptional repressor into a transcriptional activator, in complex with co-activators Mastermind-like (Maml) and p300 proteins. Notch/ CSL- dependent transcription results in the expression of *hairy/enhancer of split* (HES1-HES7) and *HES*-*related* (Hey1-2 and HeyL) genes, which are members of the bHLH Orange family of transcriptional repressors, thus inhibiting a number of genes.

Transgenic mouse models and human genetic disorders suggest that the four Notch receptors and their ligands have different functions. Logically, they should then be expected to induce different HES/Hey transcription patterns, since different HES/Hey isoforms appear to have vastly different roles in cellular specification. For example HES3 is selectively expressed in the cerebellum, HES5 is strongly upregulated in the embryo around E10 and Hey2 is preferentially expressed in the heart. It is also important to note that not all *hairy/E(spl)* family members act in the same direction. In some cases, *hairy/E(spl)* family members may even have opposing actions: Runx2 activity and osteogenesis are stimulated by HES1 [49], but repressed by Hey1/2, and HES6 promotes neurogenesis, opposing HES1-mediated repression of neuron-specific genes. Despite ample evidence suggesting different roles for individual Notch receptors and their target genes, very little data is available matching individual receptors to specific HES genes expression profiles.

Atypical Notch ligands contain IgC2 and fibronectin domains instead of EGF-like repeats and induce a different set of genes by binding to different transcription regulators, the Deltex family of transcriptional repressors. These are mostly involved in nervous system gene expression and development. Both canonical and atypical Notch ligands generate the same NICD fragments, which then go on to initiate divergent transcription mechanisms through CSL and Deltex, respectively. Canonical Notch ligands inhibit Deltex-dependent gene expression, thus inhibiting neuronal differentiation and promoting cell proliferation through the CSL/HES pathway (Fig. (**3**)).

## NOTCH INVOLVEMENT IN CARDIOVASCULAR DEVELOPMENT AND PATHOLOGY

Components of the Notch pathway have a broad developmental role, being involved in the development of the cardiovascular system but also in embryonic dorso-ventral patterning, neurogenesis, somite and limb development, myogenesis and hematopoiesis through direct cell-cell contact. A typical result of Notch action is lateral inhibition [50], by which an increase in Notch receptor activity and expression in one cell decreases receptor expression and increases ligand expression in adjacent cells. Thus, a matrix of identical cells (for example stem cells) starts to diverge when one cell responds faster than its neighbors to an external signal that activates the Notch pathway. This mechanism also helps a cell establish its position relative to other cells and maintain tissue architecture.

Transgenic mouse models have linked several Notch pathway components to vascular system development, including Jagged1, Notch1, Notch2, Notch4 and presenilin (reviewed in [51]). Mice deficient for these proteins die during embryonic development and display severe vascular abnormalities, especially Notch1/Notch4 double nulls. The pattern of Notch ligand and receptor expression during development, with some members being restricted to either endothelial or vascular smooth muscle cells, may constitute a blueprint for angiogenesis (reviewed in [52]). For example, the endothelial expression of Jagged1 is required for smooth muscle development [53], while Notch1, Notch4 and Dll4 expression, initially present in the embryo in all blood vessels, become restricted to arteries by day E13, suggesting a role for Notch in arterial/ venous specification. At the same developmental stage, Notch2 expression becomes absent in the aorta but is still present in the pulmonary artery. Notch2 KO embryos die by day E11.5 due to extensive hemorrhage, similarly to Jag1 loss-of-function phenotype. The overlap of Jag1 and Notch2 functions is also confirmed by underlying mutations in Alagille syndrome (see below).

The epithelial-mesenchymal transition, mentioned as a potential source of mesenchymal stem cells in the adult vasculature and cardiac valves, may occur as a result of Notch activation by Jag1, which represses the activation of Wnt pathway [54]. Preferential expression of Jagged1 in the endothelial cells of injured blood vessels induces high levels of Notch receptors in neighboring smooth muscle cells (Fig. (**1**)) and reduces contact inhibition [55] and cell adhesion through a reduction in cadherin levels [56]. This suggests that Jagged1 may be involved in the de-differentiation of vascular cells and the cellular proliferation phase characteristic for atherosclerosis. The balance between various Notch ligands may also regulate the osteogenic process: both Jag1 and DLL1 enhance BMP2-mediated osteogenic differentiation [57]. In contrast, Dlk1, the inhibitory ligand of Notch receptors, is highly expressed in proliferating stem cells and inhibits their full differentiation into osteoblasts and adipocytes [58].

A number of human genetic disorders resulting from mutations in Notch signaling have demonstrated the implication of Notch in the development and maintenance of the cardiovascular system. For example the Alagille syndrome is an autosomal dominant disorder associated with mutations or deletions of Jag1 gene or, in less than 1% of cases, with Notch2 mutations. Clinical symptoms are variable but typically include congenital heart defects, cholestasis, butterfly vertebrae and skeletal and facial abnormalities. The cardiovascular problems are amplified by frequent kidney abnormalities. In mouse models however, both Jag1 and Notch2 heterozygous mutations are necessary to reproduce the human Alagille phenotype [51]. The Allagille syndrome is often accompanied by tetralogy of Fallot symptoms, which include ventricular septal defects and pulmonary stenosis associated with calcified pulmonary valve. It is interesting to note that the skeletal, facial and vertebral abnormalities of the Alagille syndrome correlate with arterial calcification, suggesting that Jag1 and Notch2 may also play a role in osteoblast and bone development. A phenotype similar to the Alagille syndrome and Jagged1/Notch2 mice is present in mice homozygous for Notch target Hey2 deletion, implying the existence of transcriptional specificity for individual Notch receptors.

CADASIL (cerebral autosomal dominant arteriopathy with subcortical infarcts and leukoencephalopathy), a common form of stroke, is induced by Notch3 mutations that prevent its glycosylation and internalization [59, 60] and result in the build up of granular material in VSMC membrane, loss of intercellular adhesion and contact, and apoptosis. This induces the gradual degradation of the vascular wall, impairs blood flow through the cerebral microvasculature and generates blood leaks into surrounding tissue that result in recurrent strokes, loss of cognitive functions and death [61]. 

Finally, the strongest connection to date between Notch signaling and cardiovascular calcification was made by identifying inactivating Notch1 mutations in 2 families with a multi-generation history of bicuspid aortic valve and aortic valve calcification [44]. Wild-type Notch1 normally inhibits calcification by inducing the expression of its target genes Hey1 and Hey2, which interact with and repress the activity of Runx2/Cbfa1. Inactivating Notch1 mutations result in reduced Hey1/Hey2 expression, allowing the progression of Runx2-mediated calcification (Fig (**1**)).

## INFLAMMATORY SIGNALS AND NOTCH

A review on the role of Notch signaling in cardiovascular calcification cannot be complete without analyzing the role of Notch in macrophages, which trigger the inflammatory response and subsequent plaque formation in atherosclerosis and valve calcification (Fig. (**1**)). Juxtacrine communication between macrophages appears to be regulated by increased DLL4 and Notch3 expression and DLL4/ Notch binding, which induces a pro-inflammatory response and macrophage activation and proliferation [62]. Notch lateral inhibition mechanism could be extrapolated to a similar juxtacrine signaling between activated macrophages and VSMC or valvular myofibroblasts, inducing a phenotype change in the latter. For example, a proliferative signal could be initiated in VSMC or myofibroblasts by activating Notch signaling, while having an opposite effect in endothelial cells, where DLL4 and Notch1 inhibit cell proliferation and angiogenic sprouting [63].

In addition to juxtacrine signaling, inflammatory cytokines released by macrophages, such as IL-1B, IL-6 or TNFalpha can also regulate Notch signaling over a longer distance (Fig. (**1**)). Notch and TNFalpha/ NFkappaB pathways cooperate in several ways to induce cell proliferation (reviewed in [64]). NFkappaB can activate the Notch pathway by inducing Jagged1 expression [65], whereas IkappaBα can repress Hes1 transcription by binding to its promoter [66] and also regulates the cytoplasmic shuttling of transcriptional repressor CSL which transduces Notch signals. In turn, Notch up-regulates NFkappaB and IkappaBα transcription by sequestering transcriptional repressor CSL [67, 68]. NFkappaB targets IL-1 and IL-6 are known regulators of bone development and remodeling. Although they have been mostly thought to stimulate osteoclast proliferation, they are also strongly expressed in osteoblasts, especially in immature, proliferating osteoblasts and recently it has been shown that IL-6 enhances the osteoblastic differentiation of mesenchymal stem cells [69]. 

Another paracrine molecule released by activated macrophages is nitric oxide (NO, Fig. (**1**)). Moderate NO quantities are normally released with a vasodilatatory role by endothelial cells lining the vascular wall, but the larger amounts generated by activated macrophages can be a key factor in the mechanism of atherosclerosis [70]. NO can oxidize LDL, stimulate platelet aggregation and also induce osteoblast proliferation and differentiation [71]. In fact the roles of NO in mechanotransduction and bone remodeling, in cooperation with integrins and extracellular matrix proteins, have been well established (reviewed in [72]). NO and the reactive oxygen species it generates are also likely to have a dramatic impact on Notch receptors and ligands by disrupting some of the disulfide bonds that shape the tridimensional structure of their EGF repeats and modify their glycosylation. This in turn will impact on Notch receptor binding affinity to its ligands and other signaling molecules. Therefore the role of NO in osteoblast differentiation may be mediated in part by its modulation of Notch signaling.

## CONCLUSION

The increased risk of mortality and morbidity associated with cardiovascular calcification has lead to the design of new therapeutic strategies to prevent and even reverse this process. For example, in the case of statins, although recent clinical trials have failed to show a reduction in advanced calcific aortic stenosis, a growing body of research indicates that early use of statins may have therapeutic advantages in preventing cardiovascular calcification. 

A large number of recent publications confirm what was until recently just a hypothesis: that arterial and valve calcifications are active inflammatory diseases triggered by pro-atherogenic factors [9, 12, 73]. The molecular connections between macrophage signaling and the calcification process in arterial walls or valve leaflets are quite numerous and greatly exceed the number of publications reviewed in this article. An essential aspect of cardiovascular calcification is the multipotency of cells that are induced by inflammatory signals to proliferate and differentiate into osteoblast precursors. Notch signaling pathway is involved in this process at multiple levels: it receives input from inflammatory molecules that induce Notch-mediated cell signaling, it regulates cell proliferation and controls the differentiation of various cellular lineages including osteoblasts. Notch may have a dual role in osteoblast differentiation: contributes to inflammatory signals and stimulates early proliferation of immature osteoblasts, but also inhibits terminal osteoblastic differentiation by repressing Runx2/Cbfa1 [74]. The combined effect of these opposing signals may depend on the stage of cellular differentiation, on specific Notch receptors and ligands that are involved, and on additional signaling pathways. Much progress has been made in recent years in uncovering new connections between Notch pathway members, other pathways and osteoblast physiology and the large amount of new information underlines the complexity of this process and the necessity of establishing a rigorous time-dependent sequence of events [75]. 

Despite the complexity of cardiovascular calcification, rapid progress has been made over the past few years in understanding its intricacies and the pathways and signaling mechanisms of molecules involved. This generates the expectation that, besides preventive measures currently available such as non-specific anti-inflammatory medication and life-style changes, a new generation of mechanism-specific drugs, which can target genetic risk factors and actually reverse atheromatous plaques and calcification, will become available in the near future.

## Figures and Tables

**Fig. (1):**
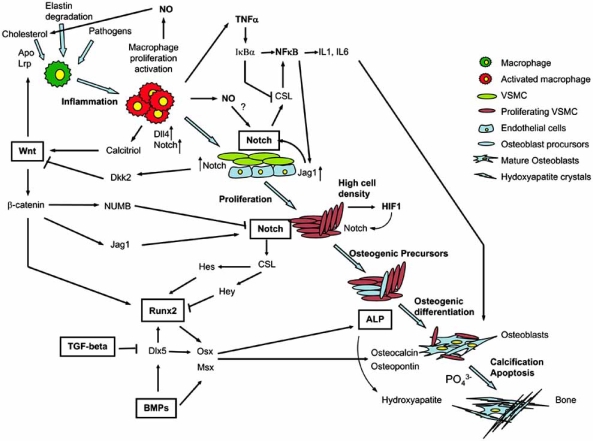
Diagram of inflammation-induced osteogenic differentiation. Changes in cellular phenotypes (open arrows) are shown in an integrated network with overlapping regulatory arrays of signal transduction (solid arrows), which form signaling hubs specific for each cell type. Major signaling pathways (Wnt, TGF-beta, BMP, Runx2-ALP) that interact with Notch are shown in boxes and major inflammatory molecules (NO, TNF-alpha, NFkappaB, HIF1) are in bold. Inflammation, initiated by fat accumulation, elastin degradation or pathogens, induces the activation and proliferation of macrophages. Activated macrophages interact with, and induce the proliferation of neighboring vascular smooth muscle (VSMCs) cells or resident stem cells (MSCs) through secreted inflammatory molecules as well as direct contact *via* Notch receptors and ligands. The balance between Notch and Wnt pathways, with regulatory contributions from inflammatory molecules, TGF-beta and bone morphogenetic proteins (BMPs), controls the proliferation/ differentiation of VSMCs or MSCs. High cell density generated by initial proliferative phase can tip the balance towards activation of Runx2/ALP and osteogenic differentiation.

**Fig. (2):**
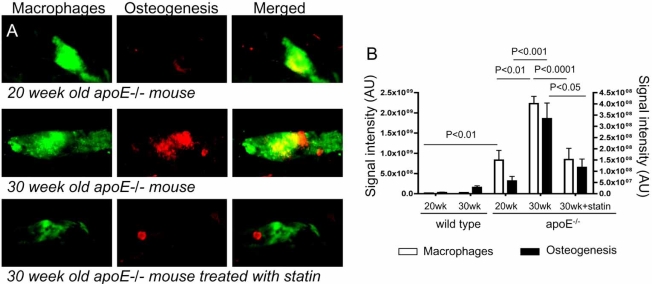
Osteogenesis associates with inflammation in early-stage atherosclerosis. A, Sequential intravital fluorescence microscopy of ApoE KO mouse carotid atherosclerotic plaques. ApoE KO mice fed a high-cholesterol diet were imaged *in vivo* at 20 weeks old, using multichannel laser scanning fluorescence microscopy for simultaneous visualization of osteogenesis (750 nm, red) and inflammation (680 nm, green). Mice were then randomized to continue with high-cholesterol diet with the addition or absence of statin for an additional 10 weeks. B, Quantification of inflammation and osteogenesis shown in Fig. 2A, demonstrating that statin treatment reduced by more than 50% areas of both inflammation and calcification at 30 weeks.

**Fig. (3):**
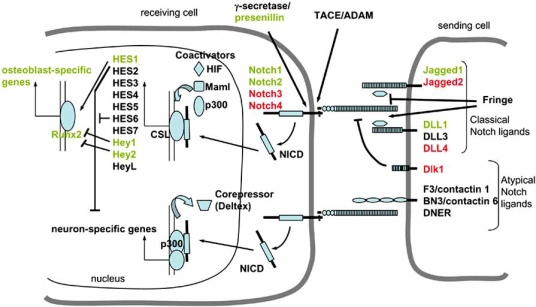
Simplified diagram of Notch signaling. Ligand binding to Notch receptors induces sequential receptor cleavage on the extracellular and intracellular sides of the membrane by TACE/ADAM and γ-secretase/presenilin proteases. The Notch intracellular domain (NICD) migrates to the nucleus and acts as a transcription regulator for cardiovascular/osteogenic or neuronal differentiation pathways. Classical Notch ligands induce signaling pathways that play a role in cardiovascular development and disease, including calcification, through CSL, HES/Hey and Runx2-regulated transcription. Notch glycosylation by Fringe favors binding of Delta-like ligands and inhibits binding of Jagged ligands. Atypical Notch ligands are involved in neuronal development and disease through Deltex-regulated transcription. The two pathways are mutually exclusive through reciprocal negative feedbacks. Proteins with a known role in cardiovascular development are shown underlined. Proteins with known roles in cardiovascular calcification are shown italics.

## Footnotes

* GR – unpublished observation
